# Treatment Options for Abdominal Rectus Diastasis

**DOI:** 10.3389/fsurg.2019.00065

**Published:** 2019-11-19

**Authors:** Majken Lyhne Jessen, Stina Öberg, Jacob Rosenberg

**Affiliations:** Department of Surgery, Center for Perioperative Optimization, Herlev and Gentofte Hospital, University of Copenhagen, Copenhagen, Denmark

**Keywords:** rectus diastasis, treatment options, physiotherapy, surgery, abdominoplasty, laparoscopy, robot assisted surgery

## Abstract

**Background:** Abdominal rectus diastasis is a condition where the abdominal muscles are separated by an abnormal distance due to widening of the linea alba causing the abdominal content to bulge. It is commonly acquired in pregnancies and with larger weight gains. Even though many patients suffer from the condition, treatment options are poorly investigated including the effect of physiotherapy and surgical treatment. The symptoms include pain and discomfort in the abdomen, musculoskeletal and urogynecological problems in addition to negative body image and impaired quality of life. The purpose of this review was to give an overview of treatment options for abdominal rectus diastasis.

**Results:** The first treatment step is physiotherapy. However, evidence is lacking on which regimen to use and success rates are not stated. The next step is surgery, either open or laparoscopic, and both surgical approaches have high success rates. The surgical approach includes different plication techniques. The recurrence and complication rates are low, complications are minor, and repair improves low back pain, urinary incontinence, and quality of life. Robotic assisted surgery might become a possibility in the near future, but data are still lacking.

**Conclusions:** Evidence on what conservatory treatment to use is sparse, and more research needs to be done. Both open and laparoscopic surgery have shown positive results. Innovative treatment by robotic assisted laparoscopic surgery has potential, however, more research needs to be done in this area as well. An international guideline for the treatment of rectus diastasis could be beneficial for patients and clinicians.

## Introduction

Abdominal rectus diastasis is a condition where the abdominal muscles are separated by an abnormal distance due to widening of the linea alba, which causes the abdominal content to bulge. The rectus fascia is intact, and the condition should therefore not be confused with a ventral hernia. Rectus diastasis can be congenital but is most commonly acquired during pregnancies and/or larger weight gain causing laxity of linea alba (1). It is a common condition in pregnancy as the incidence in the third trimester is as high as 66% whereof 30–60% persist post-partum (2, 3). Not all patients with abdominal rectus diastasis have symptoms, but when they do, treatment can be considered. The symptoms include pain and discomfort in the abdomen (4), musculoskeletal problems like pelvic instability and lumbar back pain (5), and urogynecological symptoms such as urinary incontinence, fecal incontinence, and pelvic organ prolapse (6). In addition, patients with rectus diastasis also experience lower perception of body image, lower body satisfaction, and lower quality of life compared with the general population (6, 7). Even though the condition is common, literature on treatment of rectus diastasis is sparse (5).

The purpose of this review was to give an overview of treatment options for abdominal rectus diastasis. This review is intended for general surgeons, plastic surgeons, and general practitioners. It is based on a comprehensive literature search in PubMed with search topics “rectus diastasis” and “treatment” and inclusion of the most relevant literature.

## Physiotherapy

The medical doctor may start the treatment of rectus diastasis by referring the patient to a physiotherapist for conservative (non-surgical) treatment. The success rate is questionable and not possible to state, as no identified studies report on the long-term effects of physiotherapy. We suggest that physiotherapy can begin 6 or 8 weeks post-partum, which has been chosen in some studies (8, 9). The conservative interventions are exercise, postural and back care education, external support with elastic tubular bandage or a corset, and/or aerobic exercises (5). Table 1 presents examples of conservatory approaches in published studies. The approaches are heterogeneous, and it is difficult to compare the studies. In a systematic review including four studies that assessed the effect of physiotherapy as treatment for rectus diastasis, the authors concluded that it was impossible to recommend physiotherapy in general or to recommend a specific exercise routine since the included studies both had insufficient number of patients and were heterogeneously conducted with a low level of evidence (5).

**Table 1:** Overview of published non-surgical treatments.

**Study**	**Treatment**
Sheppard (10)	In total, 16 weeks of progressive “re-education” of rectus abdominis- and transversus abdominis with exercises
Thornton and Thornton (11)	Corset or elastic tubular bandage was used, both antenatal and post-natal
Emanuelsson et al. (12)	Three months of intensive physiotherapy not further specified
Gluppe et al. (8)	In total, 16 weeks of exercise with main focus to strengthen the pelvic floor muscles
Walton et al. (13)	Six weeks of exercise, either a dynamic core stability plank exercise program or a supine core stability strengthening program
Kamel and Yousif (9)	Eight weeks of abdominal exercises, some combined with neuromuscular electrical stimulation of the rectus abdominis muscles

A recent randomized controlled trial (RCT) that included 175 primiparous women failed to show any difference in the prevalence of rectus diastasis between an exercise group completing a 16-week program and a non-exercising control group, evaluated 6 and 12 months post-partum (8). However, the presence of rectus diastasis was not an inclusion criterion. A systematic review found that physiotherapy did not lead to resolution of abdominal rectus diastasis in relaxed state, but that physiotherapy could achieve a limited reduction of the inter-recti distance during contraction of the muscles (14). In several of the included studies in this review, physiotherapy began within the first months post-partum where the diastasis might resolve naturally. Therefore, it is not possible to conclude if physiotherapy or natural resolution had an effect since the studies lacked control groups (14).

An RCT with long-term follow up compared 3 months of intensive physiotherapy with surgical treatment in patients with rectus diastasis (7). The study reported pain and restrictions in daily activities, assessed by a validated pain questionnaire, quality of life with Short Form 36 (SF-36). All patients receiving physiotherapy had significantly lower quality of life score on all eight domains of the SF-36 at baseline compared with the background population. Physiotherapy significantly improved some parts of the pain variables compared with the baseline measurements, but patients still had pain during sport and during daily activities and there was no improvement in “pain right now.” Physiotherapy significantly improved the patients' scores on four out of the eight domains in the SF-36, however, especially “bodily pain” score was still significantly lower than the background population (7).

## Surgical Treatment

Surgery can be considered when physiotherapy fails to reduce the abnormal widening of linea alba and discomforts are severe. We suggest that surgery can be considered 6–12 months post-partum since the diastasis might resolve naturally during this period (8). Surgical options include open-, laparoscopic-, and robot assisted surgery. In all described techniques, a plication of the rectus fascia is performed. During open approach, it is usually the anterior rectus fascia that is plicated, whereas during laparoscopic and robot assisted approach, it is the posterior rectus fascia that is plicated.

### Open Surgery

The success rate is overall high as most studies report a 0% recurrence rate 6 months after open surgery (7, 15–17). The most widely used open approach is a classic abdominoplasty with transverse suprapubic incision extended laterally to the anterior iliac crests to visualize the rectus muscles and the linea alba (7, 12, 15–19). Other incisions are possible as well: midline supraumbilical incision, typically used in cases of other coexisting conditions (nephrectomy and hernia) (20, 21); left suprapubic incision, extended 2–3 cm upwards; and midline ventral incision, extending from the xiphiod process to the pubic area (22, 23).

Plication can be done with or without mesh reinforcement. Mesh is often used when a coexisting hernia is present (21–23). Regarding the plication techniques, the surgeon might use single or double layer, interrupted or continuous suture, and absorbable, slowly absorbable, or permanent sutures (12, 15–19, 24). Three RCTs have compared different types of plication techniques (12, 15, 16). In the aforementioned RCT with both short-term and long-term follow up published as two articles (7, 12), the purpose was to compare physiotherapy with two different types of plication techniques in open abdominoplasty. The plication was done with either one layer of absorbable continuous sutures or two layers of slowly absorbable continuous sutures. The results showed that surgery significantly improved pain scores on the validated ventral pain questionnaire compared with physiotherapy while there was no difference between the two types of plication. There were significant improvements in the quality of life score on all eight domains of the SF-36 compared with baseline measurements, and the surgically treated patients were on the same level as the background population 1 year post-operatively. Again, there were no differences between the two types of suture techniques (12). This RCT compared surgery with physiotherapy and concluded that improvements in the two surgical treatment groups were significantly greater compared with the aforementioned improvements in the physiotherapy group (7). The second RCT compared two patient groups that were sutured with double layers of interrupted sutures using permanent or absorbable sutures, respectively (15). They reported a 0% recurrence rate in both groups assessed by CT scan 6 months post-operatively. The seroma rate was 20–30% without a significant difference between the groups, and they reported no other complications (15). The third RCT compared single layer interrupted absorbable suture plication with single layer interrupted permanent suture plication. Both groups had 0% recurrence rate at 6 months follow up assessed by imaging (16). All three RCTs had a 100% follow up rate. Based on the results from these three RCTs (12, 15, 16), surgical plication has impressive results with low complication rates, and it is not possible to recommend one plication technique over another.

Four cohort studies have assessed complications in groups of patients receiving a uniform surgical repair technique (17–19, 24). In the first retrospective cohort study, the surgeons used a continuous single layer plication of the rectus sheath (18). The study included 113 patients who were followed for a mean of 12 months (range 2–40 months). The authors reported no major complications and 18% of the patients suffered from minor complications, which was seroma formation in 8%, hypertrophic scar with need for scar revision in 8%, wound infection in 1%, and skin necrosis in 1% of the patients. In another retrospective cohort study, the plication was done in a single layer with continuous permanent suture in 22 patients (17). The follow-up was >25 months and they reported 0% recurrence rate, no hernias, and no patient discomfort, but they did not state assessment of the patient discomfort. A third retrospective cohort study, including 70 patients plicated with a single layer with continuous absorbable suture, reported a recurrence rate of 40% with a mean follow up of 64 months (range 32–109 months) (19). This high recurrence rate is an outlier, and it is not possible to say whether the authors are correct in their conclusion that absorbable sutures leads to a high long-term recurrence rate. There is a risk of attrition bias since they did not have planned follow up and did not state how the patients were chosen for the follow up in addition to only 55% of the invited patients attending the follow up. A fourth prospective cohort study included 214 patients treated with abdominoplasty with plication repair of the rectus diastasis (24). They showed significant improvements 6 months post-operatively compared with pre-operative assessments of low back pain scores and urinary incontinence, using validated questionnaires.

The recurrence rates and complication rates in open surgical treatments are in general low and the complications that occur are minor. The one study that compared surgery with physiotherapy showed better results regarding pain in the surgery group than the physiotherapy group (7). Studies have shown that open repair of abdominal rectus diastasis have improved low back pain, urinary incontinence, and quality of life in patients compared with pre-surgery (7, 24).

### Laparoscopic Surgery

The success rate for laparoscopic surgery is high, as most studies report a 0% recurrence rate 6 months after surgery (25–31). A laparoscopic technique is commonly used when diastasis and ventral hernia coexists but can also be used for treatment of abdominal rectus diastasis solely (1).

**Figure 1:**
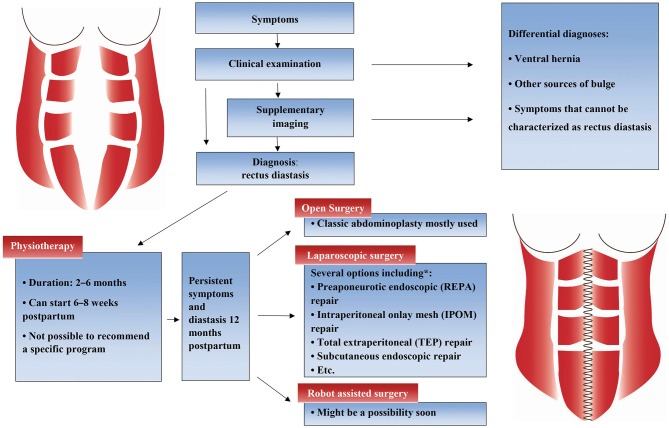
Flowchart with suggested treatment steps. ^*^: Laparoscopic techniques used for rectus diastasis repair: pre-aponeurotic endoscopic (REPA) repair (28), intraperitoneal onlay mesh (IPOM) repair (31), total extraperitoneal (TEP) repair, subcutaneous endoscopic repair (34, 36) or other endoscopic abdominoplasty techniques (29, 32, 33).

Several different placements of trocars are used, most commonly suprapubic and periumbilical (26, 29, 32–34) or suprapubic and in both iliac fossae (28, 30, 35, 36). Four studies included patients with ventral hernia, specified as umbilical, epigastric, supraumbilical, or incisional hernia (28, 33, 35, 36). As during open approach, the repair of the rectus fascia can be performed with or without mesh reinforcement. Mesh is often used when a coexisting hernia is present (27, 28, 31, 35). Single or double layer and interrupted or continuous plication technique is possible, and absorbable, slowly absorbable, and permanent suture can be used. Permanent suture has been used in most studies (26, 29, 33, 35, 36), but absorbable suture was used in 94% of 50 patients in a prospective cohort study showing 0% recurrence with a mean follow up of 23 months (28). More details about laparoscopic surgery options are shown in Figure 1.

Most studies using laparoscopic repair of rectus diastasis reported a 0% recurrence rate (25–31). The seroma formation rate in patients without a coexistent hernia has been reported to be 3–25% (26, 29, 32), hematoma rate 0% (26, 29), and infection rate 0–25% (26, 29).

Coexisting ventral hernia and rectus diastasis is a complex and significant clinical problem as well. In addition to the traditional laparoscopic intraperitoneal onlay mesh repair (25, 35), several additional techniques for surgical correction have been developed recently including endoscopic mini or less open sublay repair (EMILOS) (37) and laparoscopic intracorporeal rectus aponeuroplasty (LIRA) (38). The new techniques (37, 38) show great potential with low complication rates and less pain and might be used to correct rectus diastasis without a concomitant hernia as well. A recent study has shown the importance of detection of rectus diastasis in patients with ventral hernia. They found rectus diastasis to be a significant risk factor for recurrence when treating ventral hernia with primary sutures without mesh and they recommended checking for rectus diastasis pre-operatively (39).

### Robot Assisted Surgery

Robot assisted surgery provides new possibilities of minimal invasive treatment for specific indications. In a systematic review that compared robot assisted laparoscopic repair with open and laparoscopic repair of ventral hernias, the length of the patients' hospital stay was decreased by a mean of 3 days with robot assisted repair compared to open repair. The authors stated that it was likely that the result was due to less operative pain as fewer patients needed regional block analgesia. Robot assisted repair offered technical advantages in the closure of the hernia defect and a higher rate of defect closure was achieved with robot assisted repair compared with laparoscopic repair. However, the operating time was significantly longer for robot assisted repair compared with open repair with a mean difference of 84 min, and laparoscopic repair with a mean difference of 52 min (40).

It is likely that we will see positive results with robot assisted surgery in patients with rectus diastasis. Robot assisted surgery in the treatment of abdominal rectus diastasis is being presented at conferences as case reports and conference abstracts have been published showing promising results: low morbidity and excellent cosmetic outcome (41, 42). Unfortunately, no studies have been published that compare robot assisted surgery with open or laparoscopic surgery.

## Discussion

When a patient presents with a symptomatic rectus diastasis without a concomitant ventral hernia, the first treatment option is physiotherapy. However, the success rate of physiotherapy is questionable. Conservatory treatment includes exercise, postural, and back care education, external support with elastic tubular bandage or a corset, and/or aerobic exercises. Evidence is lacking for which conservative treatment and which exercise program to use. If physiotherapy does not lead to resolution or improvement of the rectus diastasis, surgery may be considered. Rectus diastasis can be corrected with plication performed during open or laparoscopic surgery, and both techniques have high success rates. The plication can be done in a single or double-layer, with permanent or absorbable suture, and in an interrupted or continuous way, and the literature does not imply that any plication technique is superior to another. The recurrence rates and other complications after surgical treatment are in general low, and studies have shown that repair of abdominal rectus diastasis improves various symptoms including low back pain, urinary incontinence, and quality of life.

Studies have shown a high incidence of rectus diastasis post-partum (2, 3), but there are no published articles showing how many of the women who seek medical advice. Even less is known about men with rectus diastasis. Contacting a medical doctor might be a good indicator that the symptoms are severe and have a negative impact on the patient's life. Therefore, doctors should always consider referring a patient who has rectus diastasis with symptoms to a physiotherapist for conservatory treatment. If a severe symptomatic rectus diastasis without a concomitant ventral hernia is diagnosed when women have a planned medical check a couple of weeks or months post-partum, this can be the time to refer the patient to a physiotherapist. If the patient returns after 2–6 months of conservatory treatment with persistent symptoms, a referral to a surgeon might be considered. A suggestion for treatment steps is shown in Figure 1. Some patients experience natural resolution in the first year post-partum (8), and surgery should therefore wait until at least 1 year post-partum, if possible.

Regarding physiotherapy, there is no evidence for a specific treatment method, and the choice of approach can depend on the individual physiotherapist's expertise and clinical results. Ideally, more research needs to be conducted to investigate which conservatory treatment method that is the best. So far, studies have not shown promising or significant effect of physiotherapy. Nevertheless, it is still recommendable to try conservatory treatment before considering surgery as it is not possible to determine whether the lack of evidence for conservatory treatment is due to lack of research or lack of effect. One study did show an improvement in quality of life after physiotherapy (7). More strength in the abdominal muscles likely improves symptoms in some patients, which can improve quality of life even though the rectus diastasis persists. Even though physiotherapy might not completely resolve the condition, it can improve symptoms and should always be the first line treatment.

When physiotherapy does not lead to satisfying recovery and surgery is considered in the treatment of rectus diastasis, the question is whether an open or laparoscopic approach should be performed since both approaches are associated with high success rates and low complication rates. A plastic surgeon might be chosen for the open approach with diastasis repair combined with removal of excess skin or liposuction if necessary. A general surgeon might be chosen for an open as well as a laparoscopic approach, especially when there is a coexisting hernia (14). In the future, robot assisted surgery by general surgeons might replace the laparoscopic procedure as it has potential to make the operation easier for the surgeon and still with a minimally invasive approach.

As a clinician, it is important to recognize that rectus diastasis is a significant problem and to acknowledge this patient group and their symptoms and especially their impaired quality of life compared with the background population (12). A suggestion for diagnostics and treatment strategy is presented in Figure 1. More research needs to be conducted to investigate which conservative treatment to use and to compare the surgical techniques. Preferably, an international guideline on the treatment of rectus diastasis could be compiled.

In conclusion, patients with abdominal rectus diastasis who have symptoms in addition to the bulge caused from the diastasis should be referred to a physiotherapist. Evidence is lacking for which conservatory treatment to use, and more research needs to be conducted in this area. If conservatory treatment leads to unsatisfying results, surgery can be considered. Open or laparoscopic surgery are options, and the choice of surgical technique should depend on the amount of excess skin, need for liposuction, presence of coexisting hernia, and general relative contraindications for laparoscopic surgery such as multiple previous laparotomies or peritonitis. Both types of surgery have low recurrence and complication rates. Robotic assisted surgery is being presented at conferences with promising results, and it might become a treatment method in the near future. An international guideline for the treatment of rectus diastasis could be beneficial for patients and clinicians.

## Author Contributions

MJ contributed substantially to the conception and design of the work, acquisition and interpretation of data, and the drafted work. SÖ and JR contributed substantially to the conception and design of the work, the interpretation of the data, and revised the work critically. All authors have approved the final version to be published and have agreed to be accountable for all aspects of the work.

### Conflict of Interest

JR reports personal fees from MSD, outside the submitted work. The remaining authors declare that the research was conducted in the absence of any commercial or financial relationships that could be construed as a potential conflict of interest.

## References

[1] Nahabedian M Brooks DC Rectus Abdominis diastasis - UpToDate. Available online at: https://www.uptodate.com/contents/rectus-abdominis-diastasis/print?search=rectusabdominisdiastasis&source=search_result&selectedTitle=1\sim16&usage_type$=$default&display_rank$=$1 (accessed October 22, 2018).

[2] Spitznagle TM Leong FC Van Dillen LR. Prevalence of diastasis rectiabdominis in a urogynecological patient population. Int Urogynecol J Pelvic Floor Dysfunct. (2007) 18:321–8. 10.1007/s00192-006-0143-5 16868659

[3] Sperstad JB Tennfjord MK Hilde G Ellstrom-Engh M Bo K. Diastasis recti abdominis during pregnancy and 12 months after childbirth: prevalence, risk factors and report of lumbopelvic pain. Br J Sports Med. (2016) 50:1092–6. 10.1136/bjsports-2016-096065 27324871 PMC5013086

[4] Carlstedt A Petersson U Stark B Bringman S Egberth M Emanuelsson P Abdominell rektusdiastas kan ge funktionella besvär. Lakartidningen. (2018) 115:1–3. 30457664

[5] Benjamin DR van de Water ATM Peiris CL. Effects of exercise on diastasis of the rectus abdominis muscle in the antenatal and postnatal periods: a systematic review. Physiotherapy. (2014) 100:1–8. 10.1016/j.physio.2013.08.005 24268942

[6] Keshwani N Mathur S McLean L. Relationship between interrectus distance and symptom severity in women with diastasis recti abdominis in the early postpartum period. Phys Ther. (2018) 98:182–90. 10.1093/ptj/pzx117 29228344

[7] Emanuelsson P Gunnarsson U Dahlstrand U Strigard K Stark B. Operative correction of abdominal rectus diastasis (ARD) reduces pain and improves abdominal wall muscle strength: a randomized, prospective trial comparing retromuscular mesh repair to double-row, self-retaining sutures. Surgery. (2016) 160:1367–75. 10.1016/j.surg.2016.05.035 27475817

[8] Gluppe SL Hilde G Tennfjord MK Engh ME Bo K. Effect of a postpartum training program on the prevalence of diastasis recti abdominis in postpartum primiparous women: a randomized controlled trial. Phys Ther. (2018) 98:260–8. 10.1093/ptj/pzy008 29351646 PMC5963302

[9] Kamel DM Yousif AM. Neuromuscular electrical stimulation and strength recovery of postnatal diastasis recti abdominis muscles. Ann Rehabil Med. (2017) 41:465–74. 10.5535/arm.2017.41.3.465 28758085 PMC5532353

[10] Sheppard S. The role of transversus abdominus in post partum correction of gross divarication recti. Man Ther. (1996) 1:214–6. 10.1054/math.1996.0272 11440511

[11] Thornton SL Thornton SJ Management of gross divarication of the recti abdominis in pregnancy and labour. Physiotherapy. (1993) 79:457–8. 10.1016/S0031-9406(10)60221-0

[12] Emanuelsson P Gunnarsson U Strigard K Stark B. Early complications, pain, and quality of life after reconstructive surgery for abdominal rectus muscle diastasis: a 3-month follow-up. J Plast Reconstr Aesthetic Surg. (2014) 67:1082–8. 10.1016/j.bjps.2014.04.015 24880577

[13] Walton LM Costa A LaVanture D McIlrath S Stebbins B The effects of a 6 week dynamic core stability plank exercise program compared to a traditional supine core stability strengthening program on diastasis recti abdominis closure, pain, oswestry disability index (ODI) and pelvic floor disability index scores (PFDI). Phys Ther Rehabil. (2016) 3:1–9. 10.7243/2055-2386-3-3

[14] Mommers EHH Ponten JEH Al Omar AK de Vries Reilingh TS Bouvy ND Nienhuijs SW. The general surgeon's perspective of rectus diastasis. A systematic review of treatment options. Surg Endosc. (2017) 31:4934–49. 10.1007/s00464-017-5607-9 28597282 PMC5715079

[15] Nahas FX Augusto SM Ghelfond C. Nylon versus polydioxanone in the correction of rectus diastasis. Plast Reconstr Surg. (2001) 107:700–6. 10.1097/00006534-200103000-00008 11304594

[16] Birdsell DC Gavelin GE Kemsley GM. “Staying power”–absorbable vs. nonabsorbable. Plast Reconstr Surg. (1981) 68:742–5. 10.1097/00006534-198111000-00013 6270714

[17] Asaadi M Haramis HT. A simple technique for repair of rectus sheath defects. Ann Plast Surg. (1994) 32:107–9. 10.1097/00000637-199401000-00017 8141528

[18] Kim J-K Jang J-Y Hong YG Sim HB Sun SH. Deep-plane lipoabdominoplasty in East Asians. Arch Plast Surg. (2016) 43:352–9. 10.5999/aps.2016.43.4.352 27462568 PMC4959978

[19] van Uchelen JH Kon M Werker PM. The long-term durability of plication of the anterior rectus sheath assessed by ultrasonography. Plast Reconstr Surg. (2001) 107:1578–84. 10.1097/00006534-200105000-00046 11335840

[20] Lucon M Ianhez LE Lucon AM Chambo JL Sabbaga E Srougi M. Bilateral nephrectomy of huge polycystic kidneys associated with a rectus abdominis diastasis and umbilical hernia. Clinics. (2006) 61:529–34. 10.1590/S1807-59322006000600007 17187088

[21] Matei O-A Runkel N. A novel technique of midline mesh repair for umbilical hernia associated with diastasis recti. Surg Technol Int. (2014) 24:183–7. 24526430

[22] Kohler G Fischer I Kaltenbock R Schrittwieser R. Minimal invasive linea alba reconstruction for the treatment of umbilical and epigastric hernias with coexisting rectus abdominis diastasis. J Laparoendosc Adv Surg Tech A. (2018) 28:1223–28. 10.1089/lap.2018.0018 29620963

[23] Cheesborough JE Dumanian GA Quilichini J Leyder P. Simultaneous prosthetic mesh abdominal wall reconstruction with abdominoplasty for ventral hernia and severe rectus diastasis repairs. Plast Reconstr Surg. (2016) 135:268–76. 10.1097/PRS.0000000000000840 25539311 PMC4280273

[24] Taylor DA Merten SL Sandercoe GD Gahankari D Ingram SB Moncrieff NJ. Abdominoplasty improves low back pain and urinary incontinence. Plast Reconstr Surg. (2018) 141:637–45. 10.1097/PRS.0000000000004100 29481394

[25] Palanivelu C Rangarajan M Jategaonkar PA Amar V Gokul KS Srikanth B Laparoscopic repair of diastasis recti using the “Venetian blinds” technique of plication with prosthetic reinforcement: a retrospective study. Hernia. (2009) 13:287–92. 10.1007/s10029-008-0464-z 19214651

[26] Dabb RW Hall WW Baroody M Saba AA. Circumferential suction lipectomy of the trunk with anterior rectus fascia plication through a periumbilical incision: an alternative to conventional abdominoplasty. Plast Reconstr Surg. (2004) 113:724–7. 10.1097/01.PRS.0000101508.44801.F6 14758242

[27] Kockerling F Botsinis MD Rohde C Reinpold W. Endoscopic-assisted linea alba reconstruction: new technique for treatment of symptomatic umbilical, trocar, and/or epigastric hernias with concomitant rectus abdominis diastasis. Eur Surg. (2017) 49:71–5. 10.1007/s10353-017-0473-1 28408920 PMC5368206

[28] Juarez Muas DM Preaponeurotic endoscopic repair (REPA) of diastasis recti associated or not to midline hernias. Surg Endosc. (2018) 33:1777–82. 10.1007/s00464-018-6450-3 30229321

[29] Lockwood T. Rectus muscle diastasis in males: primary indication for endoscopically assisted abdominoplasty. Plast Reconstr Surg. (1998) 101:1685–94. 10.1097/00006534-199805000-00042 9583506

[30] Siddiky AH Kapadia CR. Laparoscopic plication of the linea alba as a repair for diastasis recti - a mesh free approach. J Surg Case Rep. (2010) 2010:3. 10.1093/jscr/2010.5.3 24946321 PMC3649122

[31] Wiessner R Vorwerk T Tolla-Jensen C Gehring A. Continuous laparoscopic closure of the linea alba with barbed sutures combined with laparoscopic mesh implantation (IPOM plus repair) as a new technique for treatment of abdominal hernias. Front Surg. (2017) 4:62. 10.3389/fsurg.2017.00062 29164131 PMC5676438

[32] Chang C-J. Assessment of videoendoscopy-assisted abdominoplasty for diastasis recti patients. Biomed J. (2013) 36:252–6. 10.4103/2319-4170.113374 24225192

[33] Core GB Mizgala CL Bowen JC III Vasconez LO. Endoscopic abdominoplasty with repair of diastasis recti and abdominal wall hernia. Clin Plast Surg. (1995) 22:707–22. 8846638

[34] Correa MA. Videoendoscopic subcutaneous techniques for aesthetic and reconstructive plastic surgery. Plast Reconstr Surg. (1995) 96:446–53. 10.1097/00006534-199508000-00030 7624421

[35] Bellido Luque J Bellido Luque A Valdivia J Suarez Grau JM Gomez Menchero J Garcia Moreno J. Totally endoscopic surgery on diastasis recti associated with midline hernias. The advantages of a minimally invasive approach. Prospective cohort study. Hernia. (2015) 19:493–501. 10.1007/s10029-014-1300-2 25142493

[36] Nardi WS Busnelli GL Tchercansky A Pirchi DE Medina PJ. Diastasis recti associated with midline hernias: totally subcutaneous video-endoscopic repair. J Minim Access Surg. (2018) 14:161–3. 10.4103/jmas.JMAS_103_17 29226879 PMC5869979

[37] Schwarz J Reinpold W Bittner R Endoscopic mini/less open sublay technique (EMILOS)-a new technique for ventral hernia repair. Langenbecks Arch Surg. (2017) 1:173–80. 10.1007/s00423-016-1522-0 27766419

[38] Gomez-Menchero J Guadalajara Jurado JF Suarez Grau JM Bellido Luque JA Garcia Moreno JL Alarcon Del Agua I Laparoscopic intracorporeal rectus aponeuroplasy (LIRA technique): a step forward in minimally invasive abdominal wall reconstruction for ventral hernia repair (LVHR). Surg Endosc. (2018) 8:3502–8. 10.1007/s00464-018-6070-y 29344785

[39] Köhler G Luketina RR Emmanuel K. Sutured repair of primary small umbilical and epigastric hernias: concomitant rectus diastasis is a significant risk factor for recurrence. World J Surg. (2015) 39:121–6. 10.1007/s00268-014-2765-y 25217109

[40] Henriksen NA Jensen KK Muysoms F. Robot-assisted abdominal wall surgery: a systematic review of the literature and meta-analysis. Hernia. (2019) 23:17–27. 10.1007/s10029-018-1872-3 30523566

[41] Towfigh S Dbeis R Robotic rectus diastasis closure & umbilical hernia repair. In: Scientific Session of the Society of American Gastrointestinal and Endoscopic Surgeons. Houston, TX: Surg Endosc Other Interv Tech (2017). p. 82.

[42] Ahmad H Eckstein J Robotic assisted repair of ventral hernia and diastasis recti with rectus abdominis mobilization. In: 16th World Congress of Endoscopic Surgery. Seattle, WA: Surg Endosc Other Interv Tech (2018) p. 91.

